# Landscape genomics provides evidence of climate‐associated genetic variation in Mexican populations of *Quercus rugosa*

**DOI:** 10.1111/eva.12684

**Published:** 2018-08-31

**Authors:** Karina Martins, Paul F. Gugger, Jesus Llanderal‐Mendoza, Antonio González‐Rodríguez, Sorel T. Fitz‐Gibbon, Jian‐Li Zhao, Hernando Rodríguez‐Correa, Ken Oyama, Victoria L. Sork

**Affiliations:** ^1^ Department of Ecology and Evolutionary Biology University of California, Los Angeles Los Angeles California; ^2^ Departamento de Biologia Universidade Federal de São Carlos Sorocaba SP Brazil; ^3^ Appalachian Laboratory University of Maryland Center for Environmental Science Frostburg Maryland; ^4^ Instituto de Investigaciones en Ecosistemas y Sustentabilidad Universidad Nacional Autónoma de México (UNAM) Morelia Michoacán México; ^5^ Escuela Nacional de Estudios Superiores Unidad Morelia Universidad Nacional Autónoma de México (UNAM) Morelia Michoacán México; ^6^ Key Laboratory of Tropical Forest Ecology Xishuangbanna Tropical Botanical Garden Chinese Academy of Sciences Mengla Yunnan China; ^7^ Institute of the Environment and Sustainability University of California, Los Angeles Los Angeles California

**Keywords:** assisted gene flow, climate change, genotyping by sequencing, landscape genomics, natural selection, *Quercus*, restoration, Trans‐Mexican Volcanic Belt

## Abstract

Local adaptation is a critical evolutionary process that allows plants to grow better in their local compared to non‐native habitat and results in species‐wide geographic patterns of adaptive genetic variation. For forest tree species with a long generation time, this spatial genetic heterogeneity can shape the ability of trees to respond to rapid climate change. Here, we identify genomic variation that may confer local environmental adaptations and then predict the extent of adaptive mismatch under future climate as a tool for forest restoration or management of the widely distributed high‐elevation oak species *Quercus rugosa* in Mexico. Using genotyping by sequencing, we identified 5,354 single nucleotide polymorphisms (SNPs) genotyped from 103 individuals across 17 sites in the Trans‐Mexican Volcanic Belt, and, after controlling for neutral genetic structure, we detected 74 *F*_ST_ outlier SNPs and 97 SNPs associated with climate variation. Then, we deployed a nonlinear multivariate model, Gradient Forests, to map turnover in allele frequencies along environmental gradients and predict areas most sensitive to climate change. We found that spatial patterns of genetic variation were most strongly associated with precipitation seasonality and geographic distance. We identified regions of contemporary genetic and climatic similarities and predicted regions where future populations of *Q. rugosa* might be at risk due to high expected rate of climate change. Our findings provide preliminary details for future management strategies of *Q. rugosa* in Mexico and also illustrate how a landscape genomic approach can provide a useful tool for conservation and resource management strategies.

## 1 INTRODUCTION

Forest tree species show geographic patterns of phenotypic and genetic variation that are largely shaped by local adaptation (Langlet, 1971; Morgenstern, 1996; Savolainen, Pyhäjärvi, & Knürr, 2007; Sork, 2016). In addition to their great economic value, tree species have vast ecological importance as drivers of terrestrial biodiversity and their role in sequestering carbon (Alberto et al., 2013; Cavender‐Bares, 2016; Neale & Kremer, 2011). Recently, several biologists have raised concerns about whether tree species with their long lifespan and adaptation to local environments will be able to survive rapid climate change (Aitken, Yeaman, Holliday, Wang, & Curtis‐McLane, 2008; Rellstab et al., 2016; Sork et al., 2013). Thus, it is important to manage both plantations and natural populations with knowledge of the genetic basis of tree performance and how that variation is distributed in the natural landscape (Christmas, Breed, & Lowe, 2015; Savolainen, 2011; Sork et al., 2013). Provenance studies that compare population divergence in a range of traits, such as growth, drought tolerance, cold hardiness, and phenology, by planting seeds of different origin in one or more common gardens provide compelling evidence of local adaption that needs to be incorporated into forest management practices (Aitken & Bemmels, 2016; Bower & Aitken, 2008; Sork et al., 2013). However, when such long‐term studies are not feasible, the analysis of geographic patterns of genetic variation through a landscape genomic approach may provide an alternative source of information on adaptive genetic variation (Manel, Joost, et al., 2010; Savolainen, Lascoux, & Merila, 2013; Sork et al., 2013). This approach aims to analyze spatial patterns of genetic variation to identify evidence of local adaptation by integrating population genetic and spatial ecological modeling (Bragg, Supple, Andrew, & Borevitz, 2015; Holderegger, Kamm, & Gugerli, 2006; Joost et al., 2013; Sork et al., 2013).

Knowledge of the spatial patterns of adaptive variation in trees may be used to guide forest management decisions because it can be used to extrapolate the genetic response of trees to rapid climate change (Aitken & Bemmels, 2016; Aitken et al., 2008; Rellstab et al., 2016; Schoville et al., 2012; Sork et al., 2013). Spatially explicit predictive models would help to prioritize regions for conservation, define seed zones, and guide the choice of seed sources for reforestation based on assisted gene flow (AGF), which is the movement of individuals or propagules across the species range to facilitate faster adaptation to future predicted climates (Aitken & Bemmels, 2016). However, translating information on adaptive genomic variation into sound management decisions is still challenging (Fitzpatrick & Keller, 2015; Schoville et al., 2012) because it requires the development of accurate predictive models that consider the interaction between adaptive genetic variation and multiple environmental gradients (Aitken et al., 2008; Fitzpatrick & Keller, 2015; Schoville et al., 2012). Initial efforts of predictive models using genetic data relied on a classical species distribution modeling framework (Fournier‐Level et al., 2011; Jay et al., 2012; Sork et al., 2010). Fitzpatrick and Keller (2015) argued that SDMs have the disadvantage of not accounting for the multidimensionality of genomic variation across the landscape. Using genomic data of *Populus balsamifera* sampled in a wide geographic region as a case study, they have demonstrated that community‐level modeling frameworks (Ferrier & Guisan, 2006), such as Gradient Forests (GF—Ellis, Smith, & Pitcher, 2012) and generalized dissimilarity models (GDM—Ferrier, Manion, Elith, & Richardson, 2007), can be powerful tools to model and map turnover in allele frequencies along environmental gradients. These regression‐based models, which use nonlinear functions of environmental gradients, also offer the benefit of identifying regions of genetic and climatic similarity that could provide a basis for developing resource management practices to respond to future climate change, such as AGF (Aitken & Whitlock, 2013).

In this study, our overall objective is to utilize landscape genomic models of contemporary and future patterns of climatically associated genetic variation in the widely distributed montane oak species, *Quercus rugosa* Née (Fagaceae) and develop first‐draft management guidelines for populations facing rapid climate change. Climate change projections for Mexico indicate trends that would involve temperature increase, an overall precipitation decrease, and a change in the temporal distribution of precipitation (Karmalkar, Bradley, & Diaz, 2011; Sáenz‐Romero et al., 2009). Under this scenario, arid climates would expand in all directions and temperate forest species would be among the most vulnerable, since they inhabit the cool and humid highlands (Sáenz‐Romero et al., 2009). In fact, potential distribution models of several oak species under climate change scenarios indicated a decrease of 7%–48% in suitable area by year 2050 (Gomez‐Mendoza & Arriaga, 2007).

Given research on other oak species that reported evidence of selection on genes associated with phenology, drought resistance, and other traits (Alberto et al., 2011; Deans & Harvey, 1996; Gugger, Cokus, & Sork, 2016; Homolka, Schueler, Burg, Fluch, & Kremer, 2013; Koehler, Center, & Cavender‐Bares, 2012; Ramírez‐Valiente, Koehler, & Cavender‐Bares, 2015; Rellstab et al., 2016; Sork, Squire, et al., 2016), we designed this study to test the hypothesis that spatially divergent selection is driving differentiation among *Q. rugosa* populations in an environmentally heterogeneous region of Mexico, especially at specific loci under selection by climate. We then modeled the spatial patterns of adaptive variation across the distribution range of *Q. rugosa* in Mexico to identify the potentially most critical regions under climate change.

Our first specific objective is to identify candidate loci potentially involved in local adaptation. For this purpose, we use two conceptually different approaches. The first approach is based on the premise that loci under divergent selection show larger variation in allele frequencies among populations on the landscape than neutral genomic regions (outliers; Lewontin & Krakauer, 1973). Therefore, SNPs showing larger population differentiation (*F*_ST_) than neutral expectations may be indicative of local adaptation. These loci with significantly high *F*_ST_, however, do not point to which environmental factors might be the cause of selection (Schoville et al., 2012). Furthermore, population differentiation methods likely identify loci with strong spatial divergence and are not suitable to detect genes under selection that exhibit subtle variation in allele frequencies across the landscape (Jones et al., 2013). Thus, our second approach is to identify candidate loci that are linearly associated with climate variation across the landscape. Using the environmental association (EA) approach (Vasemägi & Primmer, 2005), we test for significant linear relationships between gradients in allele frequencies with environmental gradients to detect candidate genes under selection while controlling for population structure (Coop, Witonsky, Di Rienzo, & Pritchard, 2010; Frichot, Schoville, Bouchard, & François, 2013; Joost et al., 2007). Based on the candidate SNPs generated by the two outlier approaches, we used an annotated reference genome of *Quercus lobata* (Sork, Fitz‐Gibbon, et al., 2016) and publicly available genomic resources to identify gene models based on predicted functional annotation.

Our second objective is to use a multivariate approach to quantify the association between climatic variables, spatial variables, and genomewide genetic variants by modeling and mapping the turnover in candidate SNP allele frequencies across current and future predicted environmental gradients. We use a GF modeling framework because it models turnover directly, rather than using curve‐fitting method of GDM, which is based on distance‐based data (Fitzpatrick & Keller, 2015). This model generates informative maps of genomic information accumulated across loci of major and minor effects to identify regions of genetic and climatic similarity. We will use these findings as a basis for preliminary management recommendations for *Q. rugosa* in this region of Mexico under conditions of future climate change and as an illustration of how landscape genomic approaches can provide useful background for management and conservation strategies, especially when provenance studies may be too costly or too lengthy to utilize.

## 2 MATERIALS AND METHODS

### 2.1 Study species and sampling

*Quercus rugosa* is a white oak species (section *Quercus*) with a wide geographic distribution, from Honduras and Guatemala in Central America to Arizona, New Mexico, and western Texas in the United States. In Mexico, it can be found from the subtropics in the highlands of Los Altos de Chiapas to the temperate zones of the Sierra Tarahumara in the State of Chihuahua at altitudes ranging from 1,700 m to 3,550 m (Rzedowski, 2006; Uribe‐Salas, Sáenz‐Romero, González‐Rodríguez, Téllez‐Valdéz, & Oyama, 2008). It is one of the dominant species over much of this range, often found in monospecific stands or with other species of oak or pine. The species is most abundant along the Trans‐Mexican Volcanic Belt (TMVB), with a distribution from the western areas in the states of Jalisco and Nayarit to the eastern region in the state of Veracruz, at altitudes between 2,300 and 3,200 m (Rzedowski, 1986). The TMVB is a region with a complex geologic and climatic history. The highlands of the TMVB cross Mexico in an east–west orientation at latitude ~19°N (Metcalfe, 2006). It is an area of diverse topography and geological composition that results in a wide range of elevations and climate conditions (Gómez‐Tuena, Orozco‐Esquivel, & Ferrari, 2007; Metcalfe, 2006). The highlands forests of the TMVB are dominated by oak and pine species (Metcalfe, 2006).

**Figure 1:**
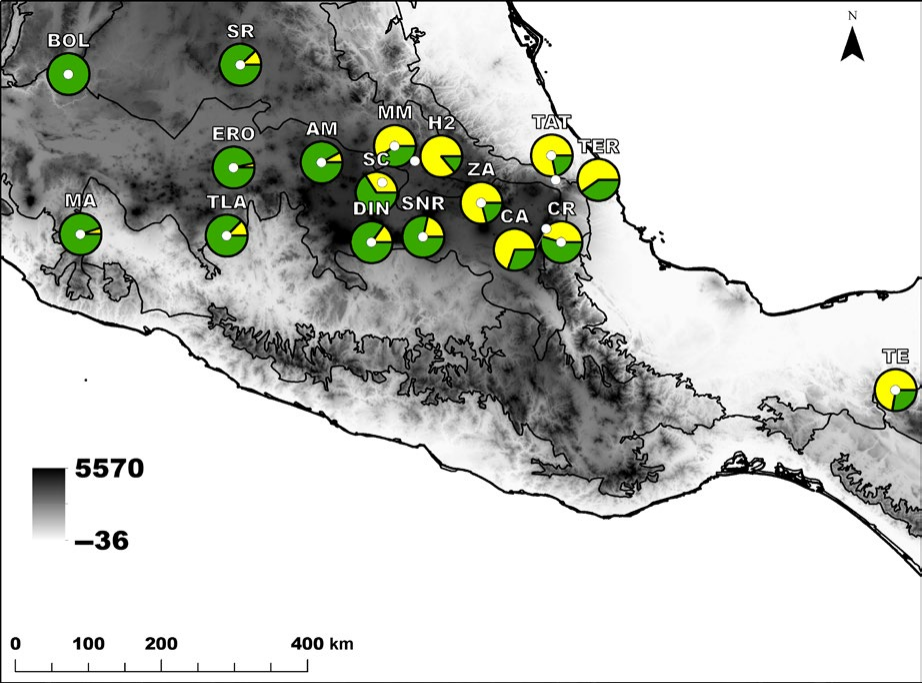
Geographic distribution of population memberships (*K* = 2) in 17 populations of *Quercus rugosa* in Mexico. Population memberships are based on Bayesian clustering method in structure, and pie charts represent population cluster assignment proportions. Shading indicates elevation gradient (with darker tones indicating higher altitude), and contour lines indicate the TMVB and neighboring physiographic regions

This study focuses on the TMVB region, and the adjacent area of Chiapas (Tenejapan) in southeastern Mexico (Figure 1). Initially, we randomly selected and sampled populations of *Q. rugosa* throughout the study area, with the criteria that they were at least 50 km apart. Here, due to DNA quality, we report on 17 natural populations from 11 states in Mexico (Supporting Information Table S1). The latitudinal and longitudinal breadth of the sampling is from about 16.7 to 21.2°N and from 92.9 to 103.2°W. Within each site, we collected leaves from 10 randomly selected individuals along a transect at least 50 m apart. Leaf samples were labeled, placed in plastic bags, kept in a cooler with ice during transport to the laboratory, and stored at −80°C until DNA extraction.

### 2.2 Laboratory procedures

Total genomic DNA was extracted from the leaves using the DNeasy Plant Mini Kit (Qiagen, Hilden, Germany) according to the manufacturer's instructions. For samples that produced final products with coloration, presumably due to unremoved secondary compounds, we repeated the extractions applying a prewash protocol (Gaddis, Zukin, Dieterich, Braker, & Sork, 2014; Li, Yang, Chen, Zhang, & Tang, 2007). Total genomic DNA was prepared for sequencing using an efficient restriction enzyme‐based approach, genotyping by sequencing (GBS) (Elshire et al., 2011), which we have modified and used for other tree species in our lab (Gugger, Liang, Sork, Hodgskiss, & Wright, 2018). Briefly, DNA was digested with a restriction enzyme, common and unique barcoded adapters with overhangs complementary to the cut site were ligated to each sample, samples were pooled in equimolar ratios, and the pooled library was PCR‐amplified and sent for Illumina sequencing. We largely followed the original GBS protocol, including the same restriction enzyme (*Ape*KI) and adapter concentration (0.036 ng/μl of each adapter). However, we pooled 48 samples per preparation instead of 96, we added adapters during the ligation step not before the restriction digest, and we added AMPure XP bead‐based size selection/purification steps after the ligation step and again after the PCR step to ensure a consistent distribution of fragment sizes between 200 and 500 bp (including adapters) among all preps. We also reduced the number of PCR cycles from 18 to 16. Final libraries were checked for the proper size distribution on an Agilent BioAnalyzer with the High Sensitivity DNA assay and quantified using a Qubit fluorometer. Samples were sent to the UCLA Broad Stem Cell Research Center for single‐end, 100‐bp sequencing on an Illumina HiSeq2000 v3.

### 2.3 Genomic data processing

Illumina reads in FASTQ format were quality filtered and demultiplexed using the “process_radtags” command in stacks 1.28 (Catchen, Hohenlohe, Bassham, Amores, & Cresko, 2013; Catchen, Amores, Hohenlohe, Cresko, & Postlethwait, 2011) to remove adapter sequence with up to two mismatches (adapter_mm), recover barcodes with up to one mismatch to the expected barcodes (r), remove any read with an uncalled base (c), discard low‐quality reads as defined by default settings (q) and trim all reads to 92 bp (t). Using BWA 0.7.12 (Li & Durbin, 2010), the filtered reads were aligned to the *Q. lobata* reference genome v0.5 (NCBI Accession LRBV00000000.1, also available at http//valleyoak.ucla.edu (Sork, Fitz‐Gibbon, et al., 2016). We used GATK 3.3 (DePristo et al., 2011) to identify SNPs in each aligned sample using a minimum confidence threshold (Phred‐scaled) of 30. We then used “VariantFiltration” and “SelectVariants” tools in GATK to exclude low‐quality variants. We applied the following filters: QD < 20.0, MQ < 40.0, MQRankSum < −12.5, and ReadPosRankSum < −8.0. We used VCftools 0.1.12b (Danecek et al., 2011) to filter the SNPs to include only diallelic sites, present in at least 95% of individuals, with minimum mean coverage depth of 5, and minor allele frequency (MAF) ≥ 0.10. We used this MAF limit to reduce the likelihood of false‐positive results due to spurious correlations. Statistics of coverage depth per locus and per sample were also performed in VCftools. SNPs were pruned in plink (Purcell et al., 2007) using the “indep” parameter. We used a variance inflation factor threshold of 2, window size in SNPs of 5, and the number of SNPs to shift the window at each step of 5.

### 2.4 Climatic variables

We downloaded 19 climatic variables from the Digital Climatic Atlas from Mexico (http://uniatmos.atmosfera.unam.mx, 926 m resolution, period: 1902–2011) and extracted values for 17 *Q. rugosa* point locations. This procedure was performed in R 3.2.0 (R CoreTeam, 2015) using the “dismo” 1.0‐12 package (Hijmans, Phillips, Leathwick, & Elith, 2015). We excluded variables that are highly correlated (ǀ*r*ǀ > 0.70) resulting in the following set of climate variables: temperature seasonality (BIO4), minimum temperature of coldest month (BIO6), precipitation seasonality (BIO15), precipitation of wettest quarter (BIO16) (Supporting Information Table S1). Some of these variables are also correlated with either latitude or longitude (Supporting Information Table S2).

### 2.5 Population structure and isolation by distance

To explore whether restricted gene flow and isolation by distance influence the genetic structure of our populations, we first estimated pairwise population differentiation using *F*_ST_ (Weir & Cockerham, 1984) and then regressed *F*_ST_/(1 −* F*_ST_) between population pairs to the log of pairwise spatial distances between populations as proposed by Rousset (1997). These analyses were performed in genepop 4.3 (Rousset, 2008). A Mantel test was performed in R using “ape” library (Paradis, Claude, & Strimmer, 2004) and 9999 permutations. We also calculated gene diversity (*H*_E_) and *F*_IS_ per population in genepop, according to Weir & Cockerham (1984).

### 2.6 Population divergence of individual loci

To identify genomic regions under spatially divergent selection, we used the Bayesian method implemented in BayeScan 2.1 (Foll & Gaggiotti, 2008) that has been recognized as the most efficient population differentiation method (De Mita et al., 2013; Lotterhos & Whitlock, 2014; Narum & Hess, 2011). We tested 5,354 SNPs using default values. In summary, prior odds for the neutral model was set to 10 and the following parameter values: 5,000 of outputted iterations, thinning interval size of 10, 20 pilot runs, pilot runs of 5,000 iterations, burn‐in length of 50,000 iterations. To decrease the chance of false positives due to multiple testing, we adopted the false discovery rate (FDR) criterion (Benjamini & Hochberg, 1995). *Q*‐values were calculated in R 3.2.0 (R CoreTeam, 2015) using “qvalue” package (Storey, 2015). We considered outliers to be SNPs with *q* < 0.05 (−log_10_ *q* > 1.3). Simulation studies have shown that BayeScan has the best performance under departure from the island model compared to other population differentiation methods (De Mita et al., 2013; Narum & Hess, 2011). Because this study species is likely to have a weak pattern of isolation by distance, this *F*_ST_ outlier analyses provide credible candidate SNPs resulting from spatially divergent selection pressures across these *Q. rugosa* populations.

### 2.7 Environmental association analysis of individual loci

As a second way of detecting SNPs potentially under natural selection for local adaptation, we tested for associations between SNPs and climatic gradients using a latent factor mixed model implemented in LFMM 1.3 (Frichot et al., 2013). This method estimates allele–environment correlations between each SNP and each variable at a time, while correcting for background population structure using latent factors. In LFMM, environmental variables are tested separately and introduced into each model as fixed effects, and the number of latent factors (*K*) is included in the model as a covariate to control for demographic history and environmental gradients not included in the study (Frichot et al., 2013). Although most EA analysis methods are prone to false negatives when demography and environment are correlated, LFMM is less prone to both false negatives and false positives (Frichot et al., 2013; Lotterhos & Whitlock, 2015) than competing methods, such as bayenv2 (Gunther & Coop, 2013), because it does not rely on a specific demographic model when accounting for population structure (De Villemereuil, Frichot, Bazin, François, & Gaggiotti, 2014; Lotterhos & Whitlock, 2015).

We used the two methods recommended by Frichot et al. (2013) to decide the range of *K*‐values to be explored in the genotype–environment association analyses. First, we used the *K*‐value from the Bayesian clustering method implemented in structure (Pritchard, Stephens, & Donnelly, 2000). We tested *K*‐values ranging from 1 to 17 and ran three independent repetitions at each *K*. We used the admixture model; the length of burn‐in period was 10,000; and the number of MCMC repetitions after the burn‐in was 100,000. We then used two approaches to decide the number of *K* that best describes our data set, the Δ*K* method of Evanno, Regnaut, and Goudet (2005) implemented in structure harvester (Earl & vonHoldt, 2011), and the rate of change in the likelihood of *K* as function of *K* as recommended by Pritchard et al. (2000). Second, we ran a principal component analysis (PCA) followed by Tracy‐Widom test (Patterson, Price, & Reich, 2006) to select the number of significant eigenvalues as one estimate of *K*. Tracy‐Widom test indicated *K *= 6 and Bayesian clustering method resulted in *K *= 2 (see Section 3, Supporting Information Figure S1). We did five independent LFMM runs using 10,000 iterations and burn‐in of 5,000. The five independent runs resulted in very similar ǀ*z*ǀ‐score estimates; the average coefficient of variation among runs was smaller than 7%. To increase the power of LFMM statistics, we calculated median ǀ*z*ǀ‐scores, which is the strength of genetic–environment association, for each locus among five runs and considered a FDR of 5% to be significant (Frichot & François, 2015). Adjusted *p*‐values (*q*) were calculated using the genomic inflation factor (*λ*) procedure described in Devlin and Roeder (1999). To confirm that the confounding effects of population structure were under control, we relied on visual observation of histograms of adjusted *p*‐values as recommended in LFMM manual (Frichot & François, 2015). Correct distributions are expected to be flat with a peak close to zero. We performed these analyses in R using scripts available in the LFMM manual. Histograms of adjusted *p*‐values for each *K* were very similar, indicating that all of them have adequately controlled for neutral genetic structure (see histograms for *K *= 2 in Supporting Information Figure S4). As the likelihood of *K* did not substantially increase in larger numbers of *K* (Supporting Information Figure S1b), we classified SNPs as candidate loci when significant (FDR < 0.05) for *K *= 2.

### 2.8 Genomic contexts of candidate SNPs

SnpEff (Cingolani et al., 2012) and BEDTools v2.25.0 (Quinlan & Hall, 2010) were used to identify positions of candidate SNPs with respect to predicted gene models on the *Q. lobata* genome (Sork, Fitz‐Gibbon, et al., 2016). The gene models were predicted by mapping contigs of the *Q. lobata* transcriptome (Cokus, Gugger, & Sork, 2015) to the genome using GMAP (Wu & Watanabe, 2005) and Sim4db (Walenz & Florea, 2011). Supporting Information Table S3 lists the genes for which candidate SNPs fall within, plus the closest upstream and downstream genes and their distances from the SNP. For genes with candidate SNPs within, Supporting Information Table S3 also lists predicted functional annotation for the genes, transferred from the carefully curated annotation of the *Q. lobata* transcriptome to identify gene annotations and orthologs with *Arabidopsis thaliana* TAIR10 gene models (Swarbreck et al., 2008).

### 2.9 Landscape of current adaptive genetic variation and future predictions

We selected GF to model current and future patterns of genetic variation. The GF modeling is a flexible model that uses a machine‐learning regression tree approach to directly model the compositional turnover in genomic variation and efficiently accommodate nonlinear gene–environment relationships (Ellis et al., 2012; Fitzpatrick & Keller, 2015). Using GF methods as described in Fitzpatrick and Keller (2015), we modeled climatic and spatial drivers of genomic variation for five SNP sets: (a) the complete SNP set (5353 SNPs), (b) the significant climate‐associated SNPs (97 SNPs), (c) the significant SNPs associated with temperature (91 SNPs), (d) the significant SNPs associated with precipitation (6 SNPs), and (e) *F*_ST_ outliers that were also associated with climate in LFMM, hereafter called double outliers (1 SNP). The SNP data were converted into minor allele frequencies per population. To ensure robust regressions, we set a filter to remove SNPs that were polymorphic in three or less than 17 populations, but only one locus of the complete SNP set was removed with this filter. For each model, we used the four climatic variables chosen for LFMM analyses as environmental predictors. As GF does not directly incorporate geographic distances, the effects of spatial processes and unmeasured environmental variation were included in the models using Moran's eigenvector map (MEM) variables as spatial predictors. MEM variables are spatial eigenfunctions calculated from the geographic coordinates of the sampling locations. This approach, which was initially named principal coordinates of neighbor matrices, was proposed by Borcard and Legendre (2002) and mathematically developed by Dray, Legendre, and Peres‐Neto (2006). We used the first half of the MEM eigenfunctions with significant positive eigenvalues as predictors of broad‐scale spatial structure and unaccounted environmental variation as proposed in previous studies (Manel, Poncet, Legendre, Gugerli, & Holderegger, 2010; Sork et al., 2013). We calculated MEM variables in R using “spacemakeR” 0.0‐5 package (Dray, 2013). We used the same parameters described in Fitzpatrick and Keller (2015) to fit GF models: 2,000 regression trees per SNP, and maxLevel = log_2_(0.368*n*)/2 and a variable correlation threshold of 0.5 to calculate conditional variable importance values as recommended (Ellis et al., 2012; Strobl, Boulesteix, Kneib, Augustin, & Zeileis, 2008). We also used default values for the proportion of samples used for training (~0.63) and testing (~0.37) each tree. The relative importance of each predictor variable and each SNP for the five GF models was assessed through weighted *R* ^2^ values. The GF turnover functions for each predictor variable included only SNPs with positive *R* ^2^ values. *R* ^2^ values can be negative due to how they are calculated, and those less than zero have no predictive power (Ellis et al., 2012). We used GF models to predict changes in allele frequencies along each environmental gradient within the geographic range of *Q. rugosa* in Mexico. For this purpose, the environmental variables of 10,000 random location points were transformed into genetic importance values using the GF turnover functions. The GF analyses were performed in R, using “gradient forests” 0.1‐17 package (Ellis et al., 2012).

To visualize the results of the GF modeling, we reduced the output of multiple transformed environmental variables (i.e., genetic importance values) into multivariate synthetic variables using PCA. The PCA was centered but not scaled to preserve the differences between genetic importance values among the environmental variables. For each of the five GF models, the first three PCs were assigned to a red‐green‐blue color palette, respectively, and visualized in geographic space. In our maps, color similarity corresponds to the similarity of expected patterns of genetic composition. We then performed a Procrustes superimposition (Gower, 1971; Jackson, 1995) on the PCAs to compare mapped genetic composition for the complete SNP set and the four candidate SNP sets. The Procrustes residuals represent the absolute distance in genetic composition between SNP sets for each point location. The Procrustes residuals were rescaled from zero to one and mapped. PCAs and Procrustes superimpositions were performed in R, using “vegan” 2.3.1 library (Oksanen et al., 2015).

To estimate vulnerability to climate change, we transformed future climate scenarios for 2080 into genetic importance values using the previous GF functions calculated for current climate. For each data point, we averaged future climate data corresponding to the representative concentration pathway 6.0 (RCP 6) scenario of greenhouse gas concentration trajectories (Fujino, Nair, Kainuma, Masui, & Matsuoka, 2006; Hijioka, Matsuoka, Nishimoto, Masui, & Kainuma, 2008) of three coupled atmosphere–ocean climate models: BCC‐CSM1.1(m) (Wu, 2012; Xin, Zhang, Zhang, Wu, & Fang, 2013; Xin, Wu, et al., 2013), CSIRO‐Mk3.6.0 (Rotstayn et al., 2012) and MIROC5 (Watanabe et al., 2010). We then calculated the Euclidian distance between current and future genetic compositions to identify geographic regions where gene–environment relationships will be most disrupted due to climate change (named as “genetic offset” in Fitzpatrick & Keller, 2015). To identify regions predicted to experience greater impacts under future environments in the lack of adaptive evolution or migration (Fitzpatrick & Keller, 2015), we mapped the genetic offsets for each SNP set.

## 3 RESULTS

The final data set included 103 individuals, 17 populations, and 5,354 SNPs, with a mean number of six individuals per population (Supporting Information Table S1). On average, samples had only 1.7% of missing data and 91.3% of the samples had <5% of missing data (the sample with the greatest number of missing loci had 16.8%). The mean depth of coverage per locus per sample was 21.8, and 88.1% of our 5,354 loci had a mean depth larger than 10× (Supporting Information Figure S2). Out of 103 samples, 72.8% had a mean depth greater than 10×.

### 3.1 Genetic diversity, population structure, and isolation by distance

The average genetic differentiation across loci and sample sites was *F*_ST_ = 0.056 with pairwise *F*_ST_ among sample sites ranging from 0.037 to 0.095 (Supporting Information Table S1). Average gene diversity was *H*_E_ = 0.364, *SD* = 0.012. The Municipio Bolaños population, which is located in the northwestern range of the sample sites, showed the lowest gene diversity (*H*_E_ = 0.327) and the highest mean pairwise *F*_ST_ (0.095) (Supporting Information Table S1). Populations exhibited a pattern of isolation by distance (*r* = 0.475, Mantel test *z* = 46.606, *p* = 0.015, Supporting Information Figure S3). Bayesian clustering implemented in structure identified *K* = 2 gene pools (Supporting Information Figure S1). The distribution of gene clusters in the landscape followed an east–west gradient (Figure 1).

### 3.2 Population divergence of individual loci

**Figure 2:**
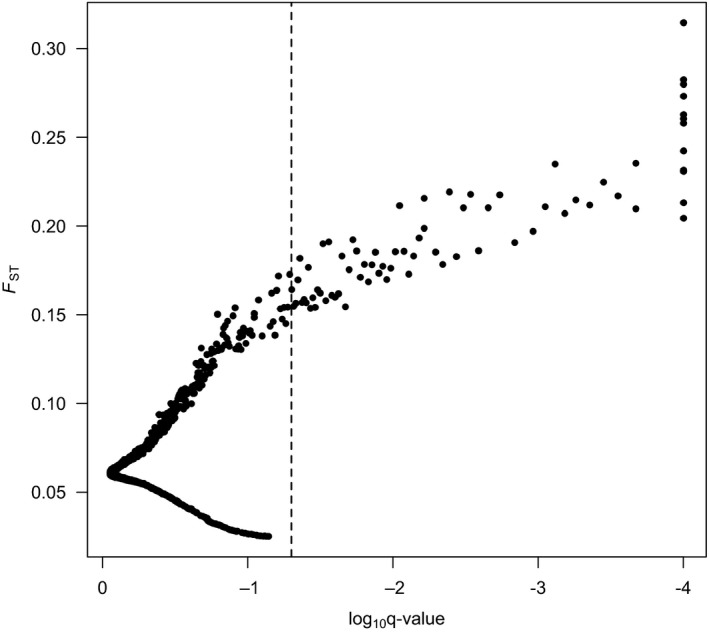
Results for the outlier *F*_ST_ test based on 17 populations of *Quercus rugosa* in Mexico. SNPs exceeding log_10_ *q* < −1.3 are classified as outliers. Values of log_10_ *q* = –4 had *q* = 0 and were truncated at –4

BayeScan identified 74 SNPs (1.4% of 5,354 SNPs) with elevated *F*_ST_ consistent with divergent selection (Figure 2). Mean *F*_ST_ of these outlier SNPs was 0.196 (*SD* = 0.035), and the range was from 0.165 to 0.314. We did not detect significantly low outlier *F*_ST_ values that would be indicative of balancing or purifying selection.

### 3.3 Environmental association analysis of individual loci

**Figure 3:**
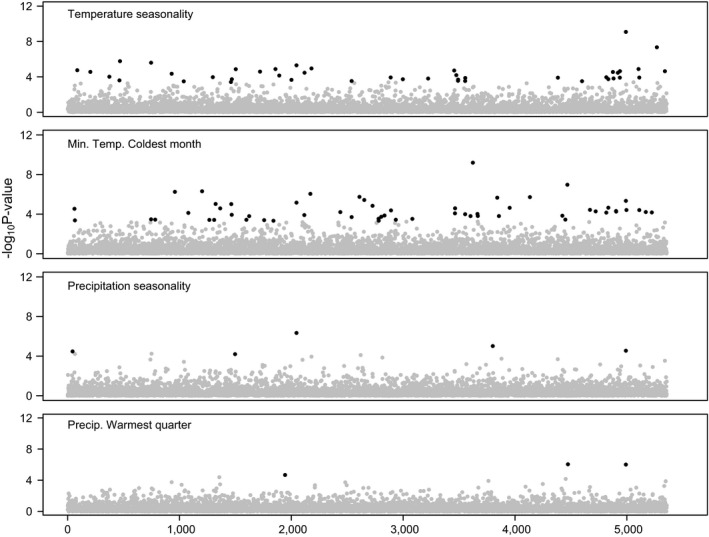
SNPs associated with temperature and precipitation variables in latent factor mixed models (LFMM) in *Quercus rugosa* in Mexico. Black dots are SNPs significantly associated with climate in *K* = 2 (adjusted *p* < 0.05). SNPs are arranged in order of position within contigs arranged by decreasing size, not according to the position in the genome

Histograms of adjusted *p*‐values were uniformly distributed and thus indicated that *K* = 2 adequately controlled for the potentially confounding effects of population structure (Supporting Information Figure S4). We found 97 SNPs (1.8% of 5,354 SNPs) that were significantly associated with climate variables and 11 of these SNPs were associated with two or three climatic variables (Figure 3). We considered only the climate variable with the strongest association (i.e., highest ǀ*z*ǀ‐score) for these SNPs in case additional EAs are due to correlation among climate variables (De Kort, Vandepitte, Mergeay, Mijnsbrugge, & Honnay, 2015). Out of the 97 outlier SNPs, 91 were associated with temperature variables and 39 of those were associated with temperature seasonality (mean ǀ*z*ǀ = 4.14) and 52 with minimum temperature of the coldest month (mean ǀ*z*ǀ = 4.02). Only six SNPs were associated with precipitation, but mean ǀ*z*ǀ‐scores were usually higher than in temperature variables. Four of these SNPs were associated with precipitation seasonality (mean ǀ*z*ǀ = 5.39) and two with precipitation of the wettest quarter (ǀ*z*ǀ = 4.50).

Combined, BayeScan and LFMM identified 170 candidate SNPs, and one SNP was identified with both methods. This SNP was associated with temperature seasonality. Climate‐associated SNPs (*n* = 97) had mean *F*_ST_ = 0.067 (range: 0.050–0.173, *SD* = 0.029), slightly higher than the background overall population differentiation (*F*_ST_ = 0.056).

### 3.4 Genomic contexts of candidate SNPs

The genomic contexts of the 170 candidate SNPs were determined based on *Q. lobata* gene models using the SnpEff variant annotator using *Q. lobata* reference genome v0.5 (Sork, Fitz‐Gibbon, et al., 2016). We predicted 71 SNPs to fall within 67 genes and 50.7% of these 67 SNPs were intron variants. Ninety‐nine SNPs were located in intergenic regions, including the SNP that was identified by both LFMM and BayeScan (Supporting Information Table S3). Out of 67 genes, 55 had annotations in *Q. lobata* transcriptome, 25 from the outlier *F*_ST_ analysis and 31 associated with climate (Supporting Information Table S3). The identified proteins represented a broad range of biological processes, as transcription (i.e., transcription factors and regulatory proteins), metabolism (protein kinases, proteins involved in ubiquitination, proteases), and ion and protein transport. Furthermore, four of these proteins are thought to be involved in response to abiotic and biotic stimuli in *Arabidopsis*, such as response to water deprivation (tetratricopeptide repeat like superfamily protein—Yuan & Liu, 2012), salt and osmotic tolerance (phosphopantothenoylcysteine decarboxylase, HAL3A gene—Kupke, Hernández‐Acosta, & Culiáñez‐Macià, 2003), oxidative and osmotic stress (mitogen‐activated protein kinase 3, MPK3 gene—Kim et al., 2011; Wang, Ngwenyama, Liu, Walker, & Zhang, 2007), drought tolerance (Kang et al., 2010), and lead resistance (Lee, Lee, Lee, Noh, & Lee, 2005) (pleiotropic drug resistance 12, PDR12 gene).

### 3.5 Landscape of current adaptive genetic variation and future predictions

We analyzed five GF models using five different SNP sets, based on the findings of our LFMM analysis of climate‐associated SNPs with single climate variables (Table 1). The GF models that explained the most variation used the nine SNP data set associated with precipitation variables in the LFMM (mean *R* ^2^ = 36.2%) and the SNP data set of 97 LFMM significant climate‐associated loci (mean *R* ^2^ = 20.4%) (Table 1). In the model using the SNP set that included all 5353 SNPs, almost 20% of the SNPs had *R* ^2^ values greater than zero (i.e., those with predictive power) and most of the SNPs with the greatest *R* ^2^ (10% upper tail of *R* ^2^ distribution) were not included in other data sets (because they are not candidates of climate association) (Table 1).

**Table 1:** Summary of the five SNP sets used to fit Gradient Forests models and parameters of model performance in 17 populations of *Quercus rugosa* in Mexico. Double outliers are *F*_ST_ outliers that are also associated with climate in latent factor mixed models (LFMM, Frichot et al., 2013)

SNP sets	Number of SNPs	# SNPs with *R* ^2^ > 0 (%)	Mean % *R* ^2^ [range]
All	5,353	986 (18.4)	15.78 [0.02–72.16]
LFMM significant loci	97	24 (24.7)	20.36 [1.46–55.02]
Temperature‐associated loci	91	22 (24.2)	13.99 [0.0003–42.31]
Precipitation‐associated loci	6	5 (83.3)	36.17 [17.51–56.00]
Double outliers	1	1 (100)	32.77

**Figure 4:**
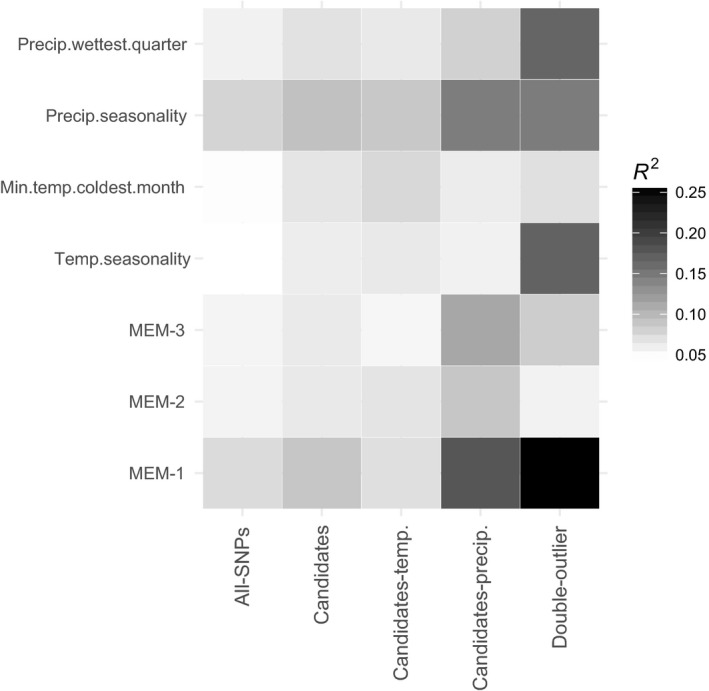
The relative importance of climatic and spatial predictors used in Gradient Forests (GF) for the five SNP sets. Darker shading indicates greater relative importance, measured as *R* ^2^ of each GF model. Candidates SNPs were those significantly associated with climate variables in LFMM. This SNP set was further separated in SNPs associated with temperature and SNPs associated with precipitation. Double outliers are SNPs that are both associated with climate and *F*_ST_ outliers

**Figure 5:**
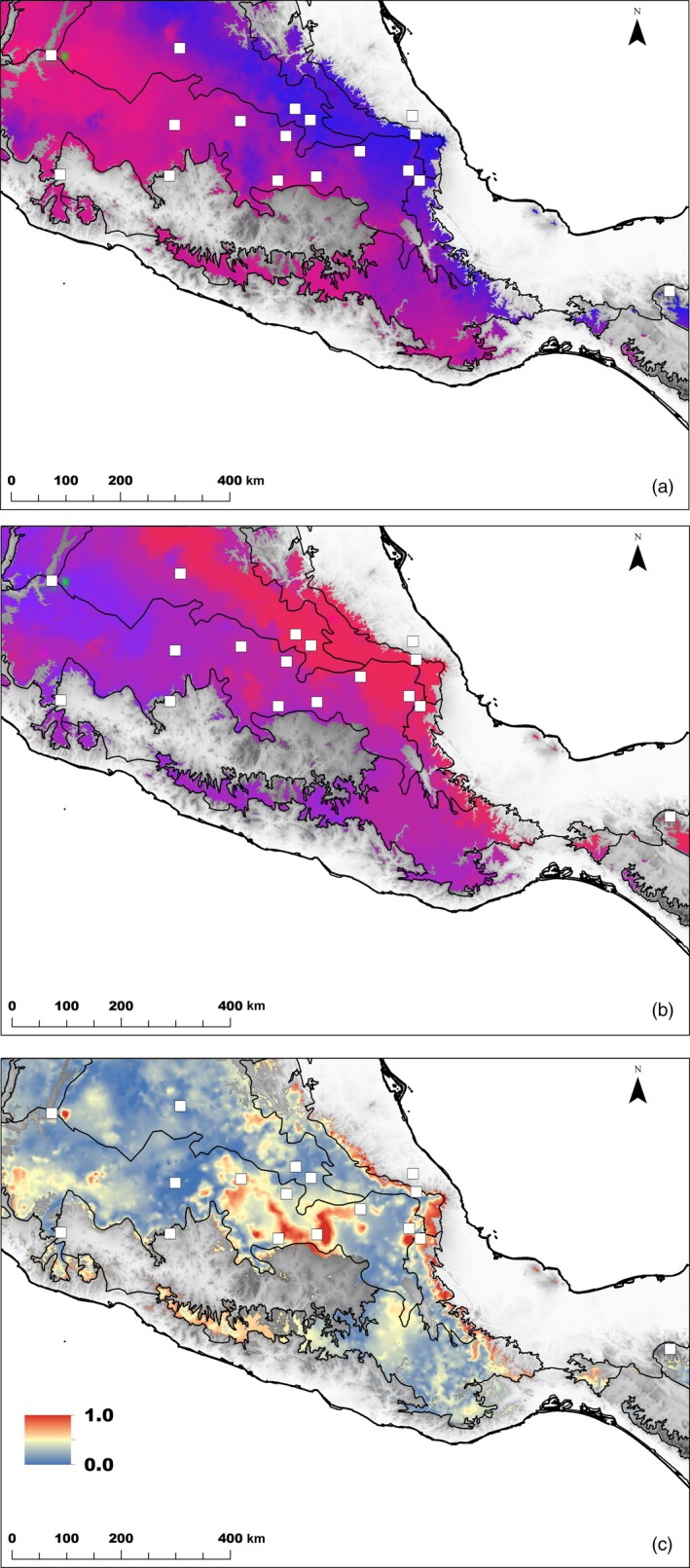
Predicted spatial turnover in allele frequencies of *Quercus rugosa* from Gradient Forests for all SNPs (a) and for SNPs associated with precipitation (b). Regions with similar colors are expected to harbor populations with similar genomic compositions. The difference between GF models (c) mapped in (a) and (b) is based on Procrustes residuals, transformed to a 0‐1 scale. White squares in (a) and (b) indicate the locations of *Quercus rugosa* populations used to fit GF models

**Figure 6:**
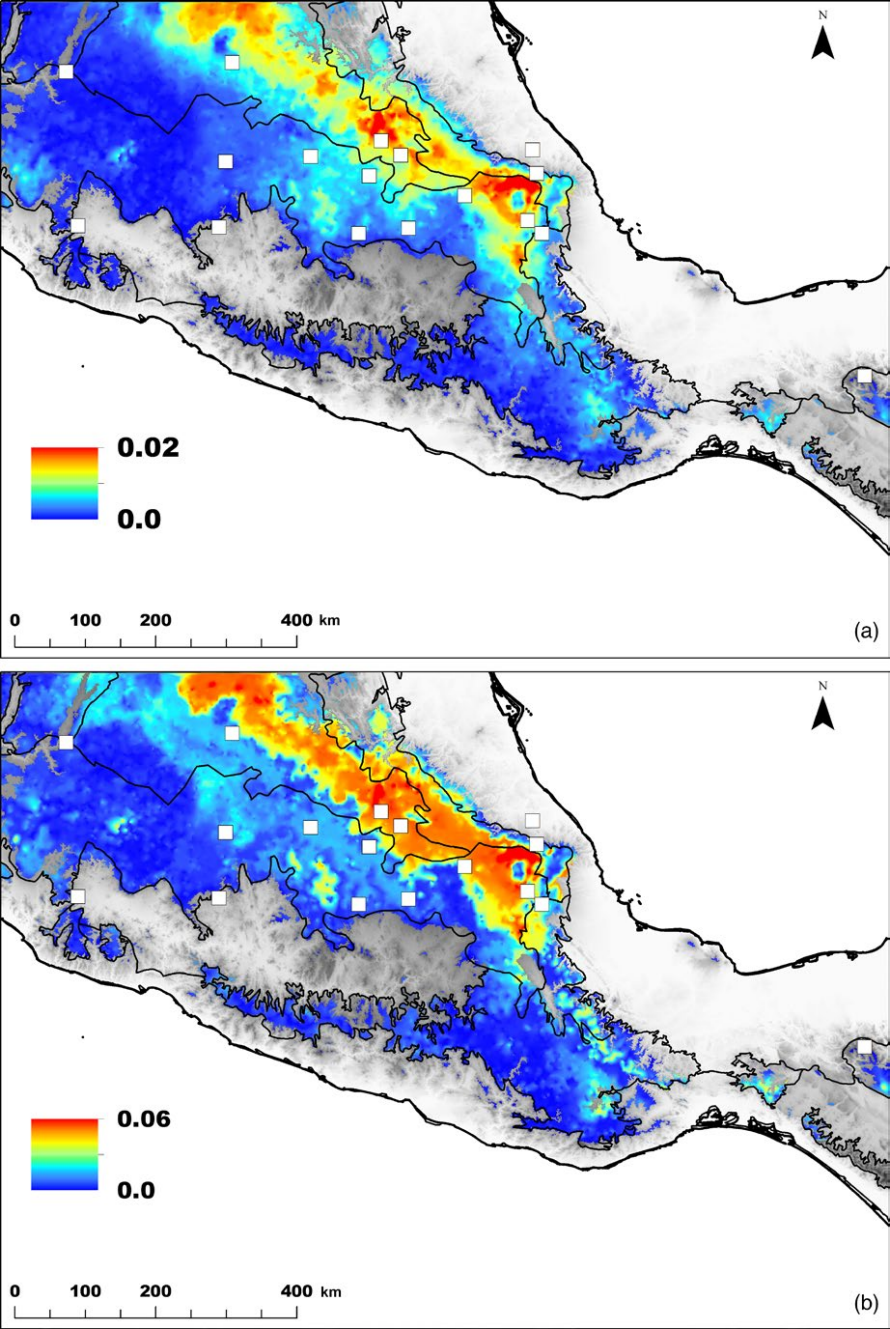
Mean predicted genetic offset for all SNPs (a) and for SNPs associated with precipitation (b) for Gradient Forests from three scenarios of 2080 climate change. Map units are Euclidian distances between current and future genetic spaces for each model. Regions with greater Euclidian distances represent large predicted genetic offset for *Quercus rugosa*

In the five GF models, precipitation seasonality and MEM‐1 spatial variable were the most important predictors (Figure 4), indicating a strong influence of the gradient in precipitation seasonality and spatial location on the turnover in allele frequency across the landscape. The strong role of MEM variables may also suggest that they have captured important unmeasured environmental predictors. In the two GF models using data sets of all SNPs and SNPs associated with precipitation, the predicted turnover in allele frequencies across the landscape was similar and followed an east–west direction (Figure 5). Although less conspicuous, the same trend was observed with the other three SNP sets (Supporting Information Figure S5). The four SNP sets of climate‐associated SNPs showed a rapid turnover in allele frequencies in eastern and central regions of *Q. rugosa* distribution, which was not evident in the data set containing all SNPs. Indeed, the difference between the pattern of genetic distribution predicted for this all SNPs set and the patterns of each of the four climate‐associated SNP sets, evaluated through the mapping of Procrustes residuals (warmer colors in Figure 5c and Supporting Information Figure S6), was small and restricted to some small areas in the eastern and central ranges of *Q. rugosa* distribution. For three SNP sets, the all SNPs, the SNPs associated with precipitation and the single outlier *F*_ST_ SNP associated with climate in LFMM, GF future predictions also indicated that northeastern populations are expected to present the greatest genetic offsets under climate change (Figure 6, Supporting Information Figure S7c). For the other two candidate SNP sets, northwestern regions also exhibited higher offsets, although with lower offsets (Supporting Information Figure S7a,b).

## 4 DISCUSSION

Our study of genomic variation in the TMVB populations of *Q. rugosa* reveals compelling evidence of geographically distributed adaptive genetic variation. Based on current genetic variation and predictive climate modeling, GF identified regions across *Q. rugosa* distribution where gene–environment relationships are most likely to be disrupted due to climate change. These geographic regions should be focal areas for further investigation in order to develop guidelines for management strategies and restoration projections.

### 4.1 Population diversity and structure

*Quercus rugosa* individuals can be assigned to two genetic clusters that showed a strong east–west gradient. This east–west pattern, which was detected in other plant and animal species occurring in the TMVB (Bryson Jr & Riddle, 2012; Parra‐Olea, Windfield, Velo‐Antón, & Zamudio, 2012; Ruiz‐Sanchez & Specht, 2014; Velo‐Antón, Parra, Parra‐Olea, & Zamudio, 2013), most likely reflects a phylogeographic signature of its orogenic history due to the different ages of the east–west regions (Mastretta‐Yanes, Moreno‐Letelier, Piñero, Jorgensen, & Emerson, 2015), given a lack of other physical barriers that could create such a pattern. The weak population differentiation (*F*_ST_ = 0.056) and high genetic diversity (*H*_E_ = 0.364) indicate high rates of historical gene flow, typical of widely distributed outcrossing woody species (Loveless & Hamrick, 1984). Population differentiation was similar to that observed in broadly distributed range‐wide populations of *Q. lobata* in California (*G*_ST_ = 0.05; Grivet, Sork, Westfall, & Davis, 2008). The small number of genetic clusters suggests extensive gene flow among populations and between clusters that would allow the spread of adaptive genetic variation across the region creating a genetic gradient, rather than numerous smaller clusters.

### 4.2 Population divergence and EAs of individual loci

Our findings provide compelling evidence of divergent selection. First, the BayeScan analysis revealed 74 outlier SNPs with these *F*_ST_ values ranging from 0.165 to 0.314, which are 2.7‐ to 6‐fold higher than the background *F*_ST_ of 0.056. This method tends to produce fewer false positives than other genetic differentiation methods (De Mita et al., 2013; Lotterhos & Whitlock, 2014). Complex demographic history could create outliers that provide false‐positive evidence of selection (De Mita et al., 2013; Lotterhos & Whitlock, 2014). Nonetheless, the large number of significant values of *F*_ST_ provides a credible set of candidate SNPs due to divergent selection pressures and local adaptation across these *Q. rugosa* populations. The EA analysis, which can be more powerful than genetic differentiation tests (De Mita et al., 2013), identified 97 candidate SNPs that are likely to represent locally adaptive genetic variation. The advantage of this approach is that the environmental factor can be identified, and for our populations, the temperature variables were more frequently significant than precipitation variables, but we add the caveat that when between‐population correlations are influenced by demographic factors such as IBD, some of the outliers may be false positives for selection (De Mita et al., 2013).

Studies of other temperate and subtropical tree species have also identified a greater proportion of SNPs associated with temperature than with precipitation (Cox, Vanden Broeck, Van Calster, & Mergeay, 2011; De Kort et al., 2014; Gugger et al., 2016; Huang et al., 2015; Jaramillo‐Correa et al., 2015). In addition, studies of high‐altitude co‐occurring species along the TMVB have found a strong and significant historical influence of temperature variables in shaping geographic distribution (Ruiz‐Sanchez & Specht, 2014; Velo‐Antón et al., 2013). But, in oaks, the number of SNPs associated with temperature and precipitation variables varies among species. In European populations of *Q. pubescens* and *Q. robur,* most of the SNPs are associated with precipitation variables (Rellstab et al., 2016), but, in *Q. petraea* (Rellstab et al., 2016) and *Q. lobata* (Gugger et al., 2016), temperature variables had most of the associations.

For a small number of SNPs, precipitation variables were important, and the strength of their associations was generally greater than SNPs associated with temperature. We are concerned that the lower number of significant precipitation‐associated SNPs in our EA tests may be due to covariance of precipitation with longitude (*r* = −0.86, Supporting Information Table S2). In general, EA models may under‐detect environmental variables that covary with neutral demographic structure (De Villemereuil et al., 2014; Lotterhos & Whitlock, 2015). Overall, both precipitation and temperature are likely important drivers of selection, but on different sets of genes within *Q. rugosa* TMVB populations.

### 4.3 Detection of candidate genes

This study identified 170 candidate loci potentially under selection, of which 67 are within functional genes annotated in *Q. lobata* transcriptome and 42 of these have previously identified orthologs in *A. thaliana* (Cokus et al., 2015). These genes are involved in a variety of physiological processes, including regulation of transcription and translation, transport of ions, proteins, metabolic and developmental processes, and response to abiotic stimuli. Evans et al. (2014) reported an enrichment of gene annotations involved in response to stimuli, regulation of transcription, and metabolic processes in *Populus trichocarpa*. Eckert, Bower, González‐Martínez, Wegrzyn, and Coop (2010) and Eckert, van Heerwaarden, et al. (2010) also found that many candidate genes identified through population differentiation or EA methods encode proteins associated with abiotic and biotic stress responses. The 67 functional genes found here are targets for future investigation of their roles in phenotypic responses to environment and fitness variation across individuals. If any of these genes can be shown to associate with fitness measurements, they could be focal genes for resource management studies.

We point out that many environmental factors other than climate, such as soil type and mineral composition, as well as numerous biotic factors such as pathogens, herbivores, or plant competition, have not been assessed and these factors could have contributed to population divergence or influenced other SNPs not identified. Moreover, because GBS protocol examines only a small portion of the genome, the goal is not to identify all genes under selection but to identify spatial patterns of adaptive genetic variation. It can be done because some of the SNPs will be located within genes under selection and others will be close to candidate genes given rapid decay of linkage disequilibrium (Neale & Savolainen, 2004; Sork, Squire, et al., 2016). Thus, landscape genomic analyses, which can capture the cumulative effects of genes under selection, will generate the spatial patterns of adaptive variation for those environmental factors that are measured.

The low level of congruence between outliers identified through population differentiation and EA tests indicates that the tests are detecting different signatures of selection (Eckert, van Heerwaarden, et al., 2010; Hancock, Alkorta‐Aranburu, Witonsky, & Di Rienzo, 2010). *F*_ST_ outlier tests are known to be very efficient in identifying strong instances of divergent selection (Narum & Hess, 2011) acting on new mutations, but has less power to detect a weak selection acting on standing variation (De Villemereuil et al., 2014; Narum & Hess, 2011) and may not detect genes that are under selection only in part of the populations (Narum & Hess, 2011). EA tests, on the other hand, have more power to detect weak selection (De Mita et al., 2013) and are better able to detect candidate genes showing subtle variation in allele frequencies across populations (Jones et al., 2013). Another explanation for this incongruence is that the climate variables we evaluated through EAs may not be the important drivers of spatial divergence at the BayeScan outlier loci. For these reasons, it is advantageous to use both analyses to detect candidate loci under selection.

### 4.4 Landscape of current adaptive genetic variation and future predictions

The five GF models indicate that precipitation seasonality represents a strong environmental driver of the turnover in allele frequencies in *Q. rugosa* in Mexico (Figure 4). Geography and unaccounted environmental gradients were also important predictors, as revealed by the greater importance of MEM‐1 variable in comparison with temperature gradients. Consequently, for all the five SNP sets, the predicted turnover in allele frequencies across the landscape followed the same east–west direction of the overall genetic structure and the precipitation seasonality gradient (Figures 1 and 5a,b). It is not surprising that spatial variables play a strong role in GF models given that most plants show spatial autocorrelation due to isolation by distance. For example, Fitzpatrick and Keller (2015) found a graduate gradient in their GF models for *P. balsamifera* and Gugger et al. (2018) found very strong spatial structure in Hawaii Island populations of *Acacia koa*, respectively. The strong spatial influence explains the similarity among GF models observed in the maps of Procrustes residuals. Nonetheless, small differences between GF models for reference and candidate loci also illustrate that recent climate environment is also shaping contemporary spatial structure.

In three models of *Q. rugosa*, our predictions for future gene–environment relationships indicate that populations in northeastern portion of *Q. rugosa* distribution in Mexico are likely to experience significant disruption (warmer colors in Figure 6 and Supporting Information Figure S7c). Considering long‐term persistence under a scenario of climate change, trees in northeastern regions are expected to be less adapted to future climate if there is no adaptive evolution or migration. Northeastern populations are likely to be more adapted to lower precipitation seasonality than the western ones. In our climate change scenarios, populations in the eastern regions could suffer from a greater increase in precipitation seasonality but also a greater decrease in precipitation of wettest quarter, while the rate of climate change is very slow in western regions. Of course, the extent to which genomic signatures detected by GBS reflect a limitation in the ability to respond to climate change is a hypothesis that still needs to be tested, not only for this study but any landscape genomic study.

### 4.5 Forest management using genomic tools

Traditionally, forest management plans have utilized provenance studies and climate modeling to select acceptable regions as transplant sources. However, the rapid rate of climate change has called for a new approach that combines spatial models of genetic variation generated by new genomic tools with climate prediction modeling to develop management and conservation strategies. For assessing the risks of climate change, both GF and other spatial models (Fitzpatrick & Keller, 2015; Razgour et al., 2017; Rellstab et al., 2016) provide statistical methods to develop those strategies.

In this study, given that future climate might change drastically in some parts of the species range, we explore whether forest management of *Q. rugosa* might benefit from AGF (Aitken & Whitlock, 2013). For example, in the northeastern region, it might be appropriate to bring in seed from western regions where seed sources likely include preadapted genotypes to future precipitation conditions. Because AGF is not without its risks (Aitken & Bemmels, 2016), we would advise using a composite seed sourcing with a mix local seeds, preadapted to a smaller precipitation of wettest quarter, with translocated seeds, preadapted to a broader precipitation seasonality. We caution, however, that, while this sample design is sufficient to illustrate how a landscape genomic/climate modeling approach could identify regions of concern, it is not sufficiently fine scale to detect the heterogeneity in genetic variation and climate niches across the species range. Thus, before finalizing specific plans for this species, or any focal species, we recommend increased sampling that includes more localities within the region(s) of concern. In addition, it would be valuable to conduct focal seedling experiments with genotypes from different regions exposed to varying water and temperature treatments to see how robust local seedling populations are tolerating environmental changes and to see whether the proposed transplanted genotypes would survive in the new region as a way to ground‐truth the recommended strategies. Information about the quantitative genetic variation in phenotypes is underway for this species, and future work can explore how phenotypic variation aligns with spatial patterns of genomic variation. When possible, the inclusion of information about quantitative genetic variation will provide a useful complement to the approach of this study by combining phenotypic information from the same populations grown in common gardens with spatially explicit genotypes of landscape genomic studies (Sork, 2017; Sork et al., 2013). For example, the use of provenance studies and spatially associated neutral genetic variation to generate “seed zones,” an established approach to forest management (e.g., Westfall & Conkle, 1992), can be enhanced by landscape genomic tools that identify adaptive variation to select seed sources for future forest management. Such an approach will be particularly helpful if rapid climate change creates the need for AGF (Aitken & Whitlock, 2013).

## 5 CONCLUSIONS

This study demonstrates that natural populations of *Q. rugosa* in TMVB exhibit geographic patterns of genetic structure that are likely the outcome of spatially divergent selection, as well as demographic history. Such information provides a first‐round assessment of regional patterns of adaptive genetic variation that will help develop a conservation and/or management plan for the preservation of oak forests in this region. For example, given the current distribution of putatively adaptive variation and future climate change, our analysis indicates certain regions of the species range that may be most at risk with rapid climate change. Analyses such as the ones presented here provide a basis both for additional sampling to create a more fine‐scale picture of the distribution of adaptive genetic variation and also for specific experiments that could assess the sensitivity of seedlings transplanted into current climate regimes in the anticipation that they will be adapted to future climate. These experiments could suggest whether current genetic variation is sufficient to tolerate future climate conditions or whether practices, such as AGF, would effectively enhance the persistence of ecosystems associated with tree species, such as this Mexican oak species. Our study presents compelling evidence that portions of the species range will be at risk under future climate change scenarios because underlying adaptive genetic variation may no longer be optimal for future climates and that conservation or management strategies of *Q. rugosa* should take this risk into account.

## CONFLICT OF INTEREST

None declared.

## AUTHOR CONTRIBUTIONS

AG‐R, PFG, and VLS designed the study with input from KO; PFG, JL‐M, and J‐LZ led the field and laboratory research; SF‐G and KM conducted bioinformatics; HR‐C prepared the maps; KM and VLS led data analysis and manuscript preparation with input from PFG, AG‐R, and SF‐G.

## DATA ARCHIVING STATEMENT

Data available from the Dryad Digital Repository: https://doi.org/10.5061/dryad.b56tm0t.

## Supporting information


** **

** **
