# Supplementary material for: Landscape genomics provides evidence of climate‐associated genetic variation in Mexican populations of Quercus rugosa
Source: Evol Appl. 2018 Aug 31;11(10):1842–58. doi: 10.1111/eva.12684 (PMC6231481; doi:10.1111/eva.12684)
Supplement: Supplementary file 1 [file EVA-11-1842-s001.pdf]

Supporting Information

Landscape genomics provides evidence of climate-associated adaptive genetic variation in Mexican populations of *Quercus rugosa.*

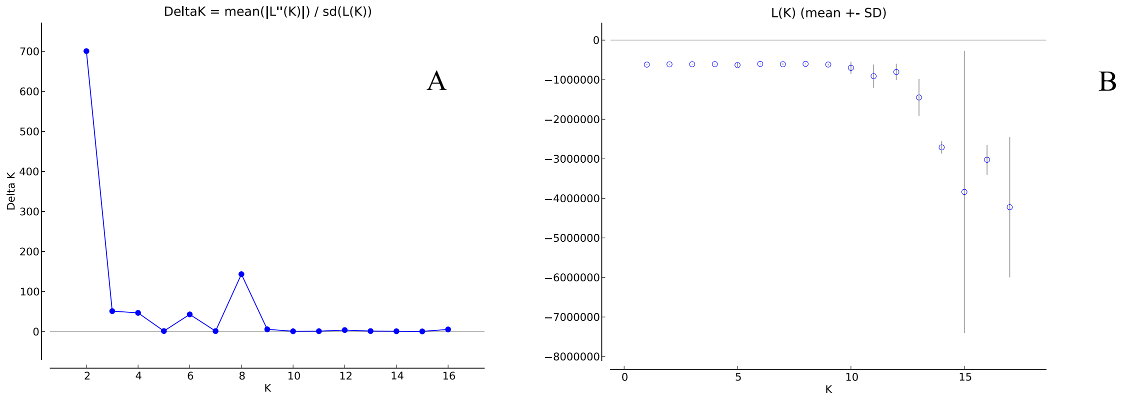

Fig. S1 Results of the STRUCTURE analysis in *Quercus rugosa.* (A) Magnitude of ΔK as a function of K, with K-values ranging from 1 to 17. (B) Likelihood of K for each value of K.

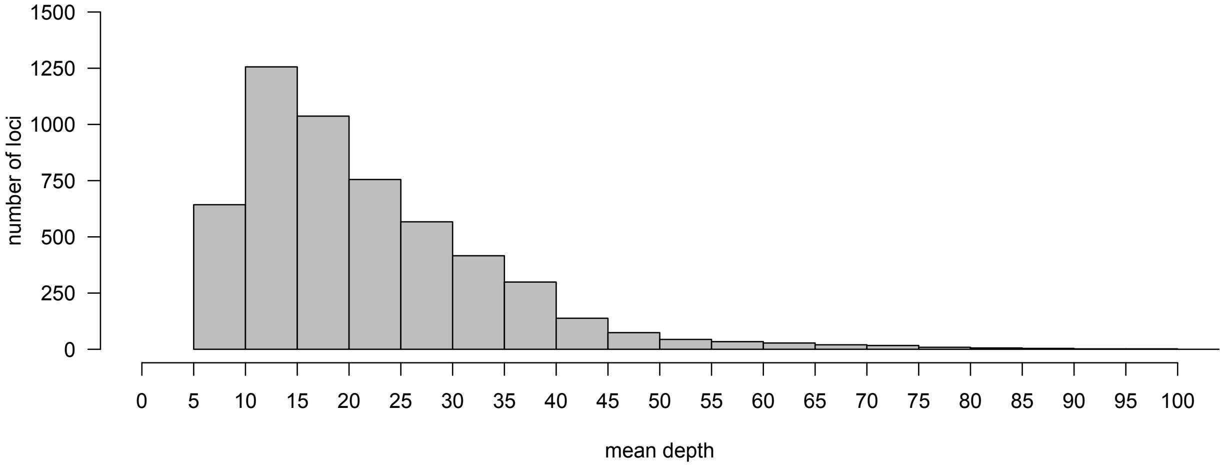

Fig. S2 Distribution of mean coverage depth per locus. Only mean depth above 5× was included.

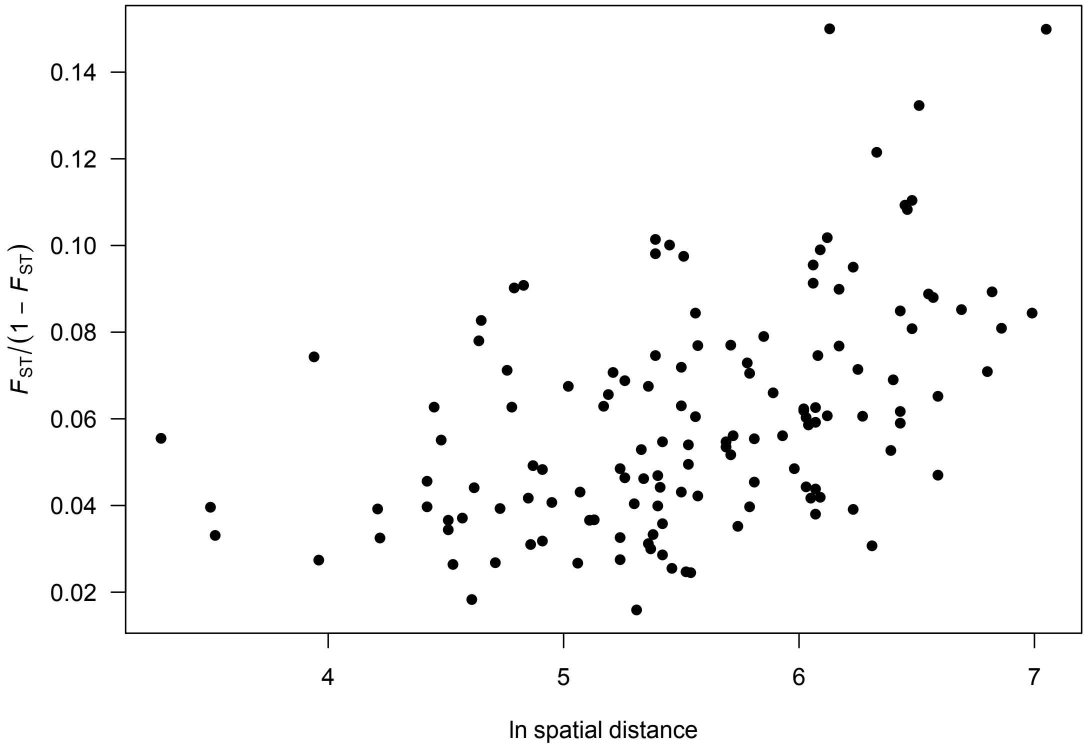

Fig. S3 Scatter plot of genetic vs. geographic distance in 17 populations of *Quercus rugosa* sampled in Mexico (r= 0.475, Mantel test z = 46.606, p = 0.015).

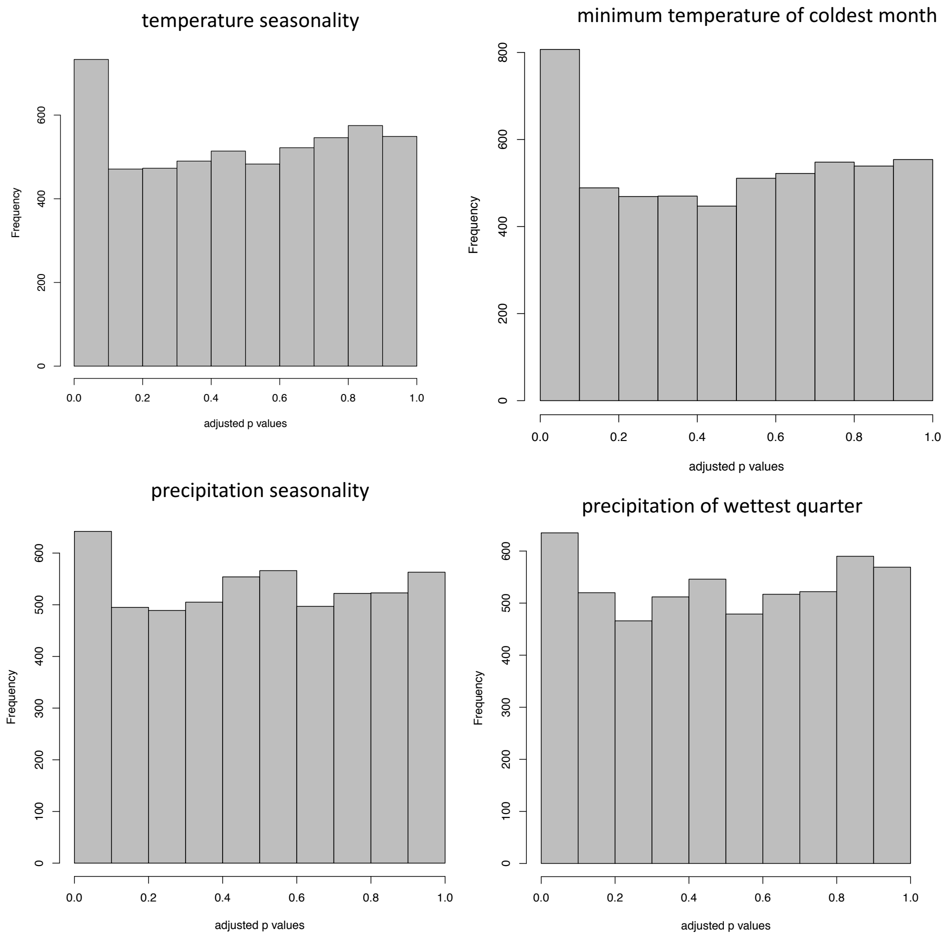

Fig. S4 Histograms of adjusted *p*-values of LFMM analyses in *Quercus rugosa*, for *K*=2 and four climatic variables.

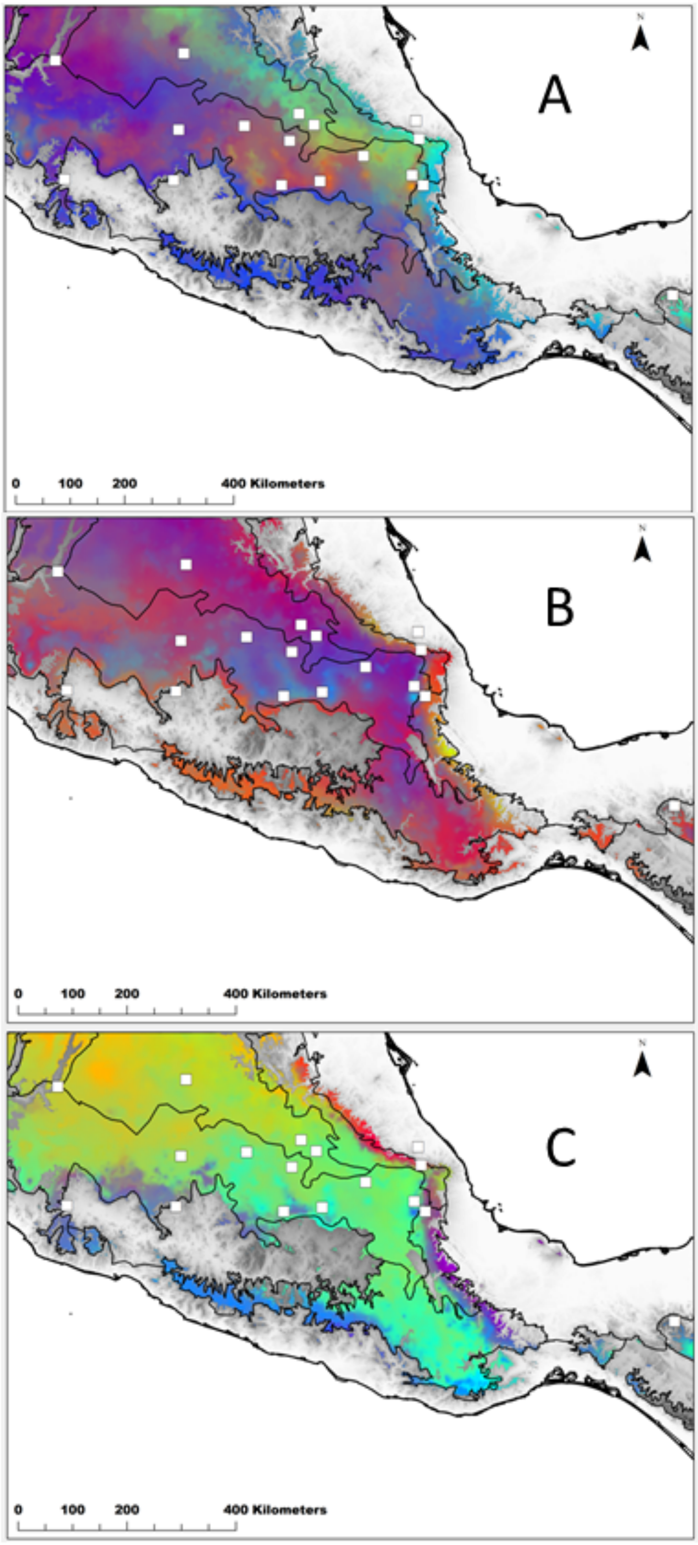

Fig. S5 Predicted spatial turnover in allele frequencies from Gradient Forests (GF) for LFMM candidates (A), for SNPs associated with temperature (B), and for F_{ST} outlier SNPs associated with climate (double-outliers) (C). Regions with similar colors are expected to harbor populations with similar genomic compositions. White squares indicate the locations of the 17 *Quercus rugosa* populations in Mexico used to fit GF models.

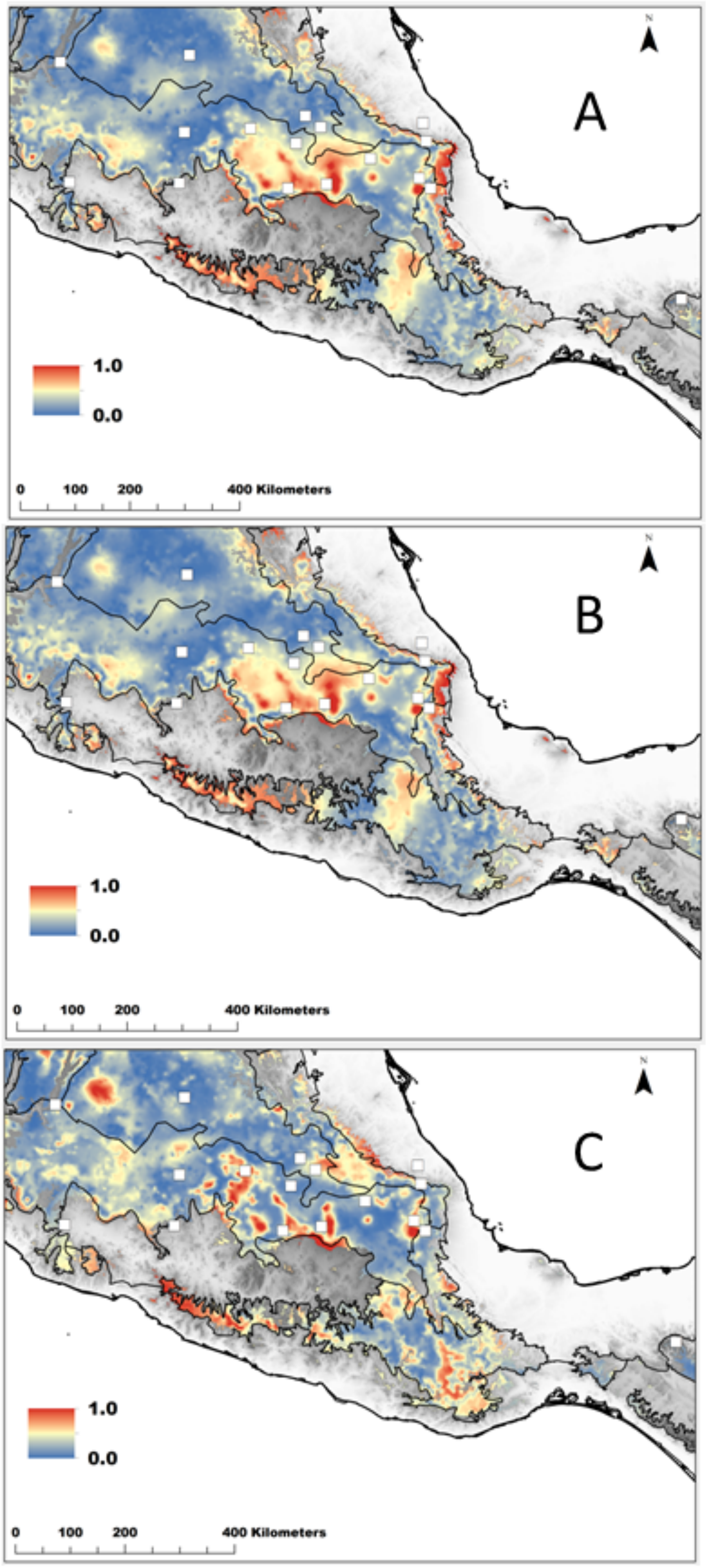

Fig. S6 Difference between the Gradient Forests model fitted to the complete SNP set and the models fitted with LFMM candidates (A), the SNPs associated with temperature (B), and the F_{ST} outlier SNPs associated with climate (double-outliers) (C). Differences are based on Procrustes residuals, transformed to a 0-1 scale. White squares indicate the locations of 17 *Quercus rugosa* populations used to fit GF models.

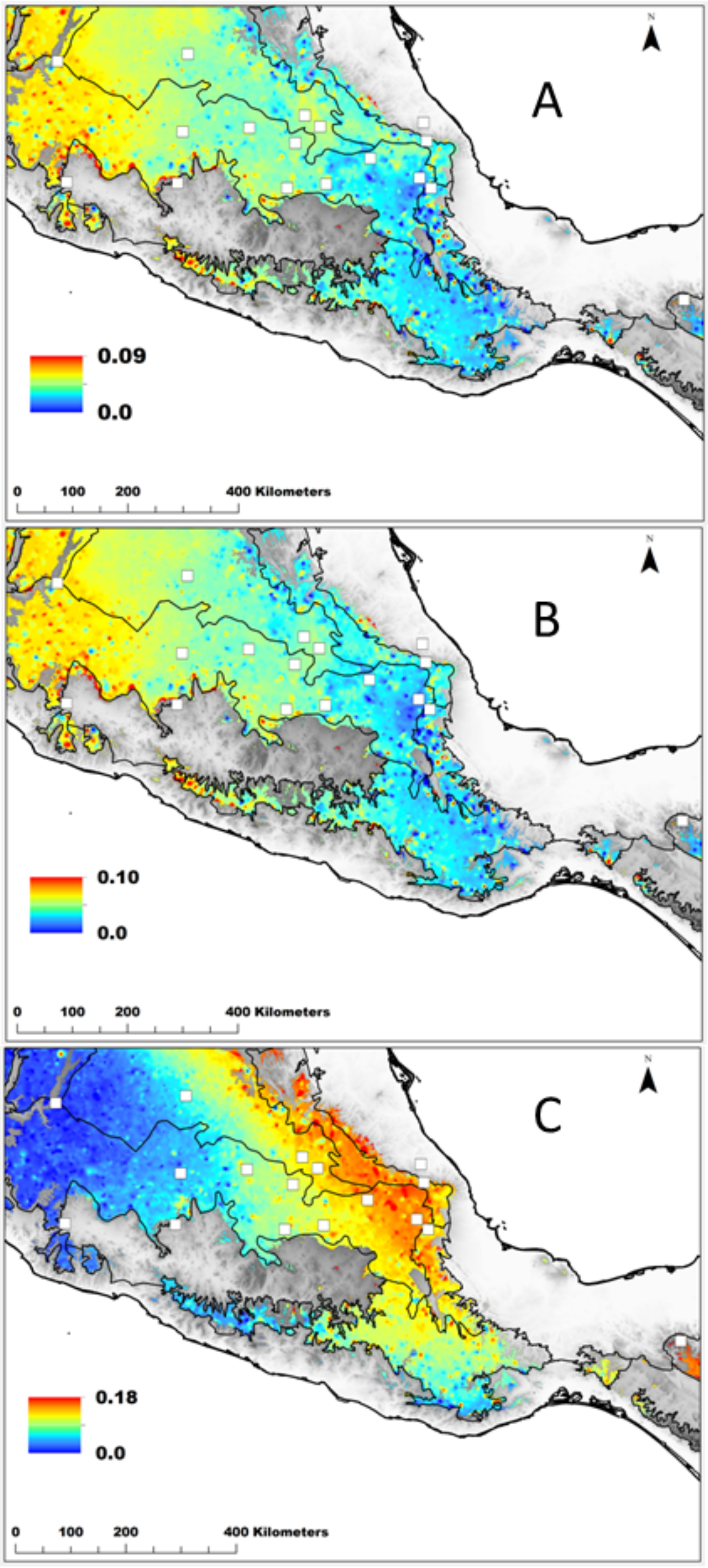

Fig. S7 Mean predicted genetic offset of *Quercus rugosa* for LFMM candidates (A), for SNPs associated with temperature (B) and the F_{ST} outlier SNPs associated with climate (double-outliers) (C) for Gradient Forests from three scenarios of 2080 climate change. Map unities are Euclidian distances between current and future genetic spaces for each model. Regions with greater Euclidian distances represent large predicted genetic offset.

Table S1 Geographic locations and climatic data of 17 *Quercus rugosa* sampling sites in Mexico, mean pairwise F_{ST} per population, gene diversity (H_E) and inbreeding coefficient (F_{IS}). Climate variables are: temperature seasonality (Temp. Season.), minimum temperature of coldest month (Min. Temp.), precipitation seasonality (Precip. Season.), precipitation of wettest quarter (Precip.).

Population name	Code	State	Sample size	Latitude	Longitude	Temp. Season.	Min. Temp.	Precip. Season.	Precip.	Mean F_{ST}	H_E	F_{IS}
Amealco	AM	Querétaro	6	20.0	-100.0	164.21	2.05	96.64	479.68	0.050	0.362	0.009
Mpio. Bolaños	BOL	Jalisco	5	21.1	-103.2	272.60	5.11	113.59	550.37	0.095	0.327	-0.017
Cacahualco	CA	Veracruz	7	19.2	-97.3	107.03	-2.40	81.34	952.20	0.049	0.370	0.035
Cerro de la Malinche	CR	Tlaxcala	5	19.1	-97.1	173.39	6.79	73.41	911.37	0.069	0.355	-0.022
Los Dinamos	DIN	DF	8	19.1	-99.4	132.44	1.40	94.06	736.61	0.056	0.358	0.009
Erongarícuaro	ERO	Michoacán	6	20.0	-101.1	217.65	4.83	100.75	411.81	0.055	0.357	0.097
Alfahayucan (Hidalgo2)	H-2	Hidalgo	9	20.1	-98.9	174.55	2.19	73.45	286.22	0.078	0.364	0.040
Mazamitla	MA	Jalisco	5	19.2	-103.0	159.54	14.07	105.13	530.25	0.056	0.359	0.188
Mineral del Monte	MM	Hidalgo	6	20.2	-99.1	205.27	3.84	72.30	199.51	0.037	0.383	0.140
Santa Catarina	SC	Edo. Méx	6	19.8	-99.3	192.64	2.19	88.60	464.21	0.047	0.371	0.015
San Nicolás de los Ranchos	SNR	Puebla	5	19.1	-98.8	161.39	2.41	91.24	502.79	0.048	0.367	-0.095
Santa Rosa	SR	Guanajuato	5	21.2	-101.0	253.27	3.55	87.92	278.39	0.052	0.370	0.021
Tatatila (veracruz1)	TAT	Veracruz	8	20.1	-97.2	298.28	13.92	48.41	592.53	0.046	0.378	0.233
Tenejapan	TE	Chiapas	3	17.2	-93.0	167.59	10.79	41.94	815.89	0.069	0.358	0.016
Terranate	TER	Tlaxcala	8	19.8	-97.2	251.68	10.00	54.48	1054.78	0.051	0.369	0.019
Tlalpujahua	TLA	Michoacán	5	19.1	-101.2	175.92	13.48	109.15	709.23	0.041	0.370	-0.050
Zacatepec	ZA	Puebla	8	19.5	-98.1	160.09	0.87	78.61	351.88	0.057	0.369	0.144

Table S2 Correlation of spatial and climate variables associated with 17 localities *of Quercus rugosa* (see Figure 1).

	Precip. seasonality	Precip. wettest quarter	Temp. seasonality	Min. temp. coldest month	Latitude × longitude	Longitude	Longitude × longitude	Latitude × latitude
Precipitation, wettest quarter	-0.262	1	-	-	-	-	-	-
Temperature seasonality	0.272	0.105	1	-	-	-	-	-
Min. temperature, coldest month	-0.236	0.251	-0.553	1	-	-	-	-
Latitude × longitude	0.178	-0.096	0.098	-0.017	1	-	-	-
Longitude	-0.859	0.429	0.140	-0.022	-0.293	1	-	-
Longitude × longitude	0.001	0.183	-0.154	0.440	-0.786	0.000	1	-
Latitude	0.200	-0.476	-0.628	-0.107	0.208	-0.548	-0.276	1
Latitude × latitude	-0.106	-0.049	-0.263	0.148	-0.920	0.087	0.780	0.000
